# Complete Mitogenome of the *Triplophysa bombifrons*: Comparative Analysis and Phylogenetic Relationships among the Members of *Triplophysa*

**DOI:** 10.3390/genes14010128

**Published:** 2023-01-02

**Authors:** Xinyue Wang, Yong Song, Haoyang Xie, Fangze Zi, Shengao Chen, Site Luo

**Affiliations:** 1 College of Life Science and Technology, Tarim Research Center of Rare Fishes, Tarim University, CN-0997, Alar 843300, China; 2 School of Life Sciences, Xiamen University, Xiamen 361102, China

**Keywords:** *Triplophysa bombifrons*

## Abstract

In the last decade, the phylogenetic relationships within the genus *Triplophysa* have become controversial, due to a lack of molecular data. The mitochondrial genome plays a vital role in the reconstruction of phylogenetic relationships and in revealing the molecular evolution of bony fishes. Herein, we obtained the complete mitogenome of *Triplophysa bombifrons* via HiFi reads of the Pacbio Sequel II system and DNBSEQ short-reads. We compared all available mitogenomes of the *Triplophysa* genus and reconstructed the phylogeny of Nemacheilidae, based on the mitogenomes, using maximum likelihood (ML) methods. The results show that the complete mitogenome sequence of *T. bombifrons* was circular and 16,568 bp in length, including 13 protein-coding genes (PCGs), 22 transfer RNA (tRNA), 2 ribosomal RNA (rRNA), and a typical control region (D-loop). The most common start codons were ATG, except for *cox1*, and TAA/TAG were the stop codons for all PCGs. In total, 677 SNPs and 9 INDELs have been found by comparing the sequence divergence between this study and previous reports. Purity selection was found in all PCGs. Phylogeny was inferred by analyzing the 13 PCGs and the concatenated nucleotide sequences of 30 mitogenomes. The phylogenetic analyses based on the nucleotides of the 13 PCGs supported the assumption that the *Triplophysa* genus can be divided into 4 main clades and demonstrated that *T. bombifrons* and *T. tenuis* are closely related species for the first time. This study laid the foundation for further study on the mitogenome and phylogeny of Nemacheilidae fishes.

## 1. Introduction

As the world’s largest clade of primary freshwater fishes, the order Cypriniformes is divided into two superfamilies: Cyprinoidea (carp-like fishes) and Cobitoidea (loach fishes) [1]. The genus *Triplophysa* (Cobitoidea: Nemacheilidae) is a species-rich group that is an important component of the ichthyofauna of the Qinghai-Tibetan Plateau (QTP). The rapid and persistent elevation of the QTP is considered a major reason for the origin and diversity of this genus. Due to its strong adaptability to extreme environments, species in the *Triplophysa* genus are widely distributed in the QTP. This genus represents an ideal system by which to address questions about past climatic and geological events and their impacts on current biodiversity. Due to the morphological plasticity of this genus, traditional taxonomy cannot accurately distinguish all species, particularly in the case of morphologically similar and related species [2,3,4,5,6,7,8,9,10,11,12,13,14,15,16,17,18,19,20].

**Figure 1:**
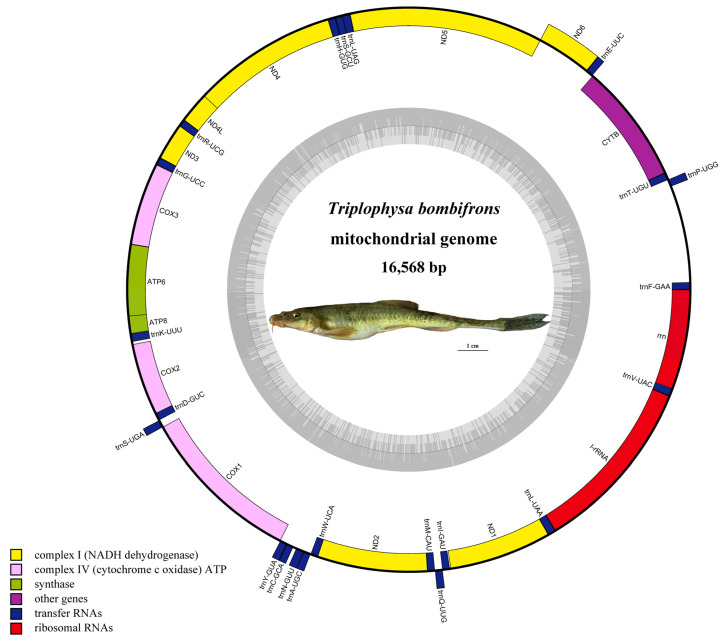
The circular map of the *T. bombifrons* mitogenome. The outer circle and inner circle represent the H-strand and L-strand, respectively. The GC and AT contents were plotted in the dark and light regions in the inner grey circle, respectively.

The mitochondrion is a very important organelle in the eukaryocyte that exists in nearly all the bionts. Mitochondria are involved in energy metabolism, aging, apoptosis, and disease regulation [21]. Close circular double-stranded mitochondrial DNA represents a good molecular marker in systematic studies due to its simple structure, fast evolution, and high copy speed, along with its easy separation and purification. [21]. Over the last decade, more than 20 *Triplophysa* mitogenomes have been reported [3,4,5,6,7,8,9,10,11,12,13,14,15,16,17,18,19,20]. Although some groups of *Triplophysa* have been addressed phylogenetically, a comprehensive phylogenetic analysis has never been presented. *T. bombifrons* is a bony fish with a narrow distribution in China. On the one hand, water hubs, environmental pollution, overfishing, and other human activities have contributed to its endangered status. On the other hand, the endangerment of indigenous fishes has intensified under degraded environments such as alpine climatic conditions and salinized water features. One mitogenome of *T. bombifrons* has been reported, which was collected from Balkhash Lake in China [17]. According to the contents of the zoography and the distribution surveys over the last ten years, *T. bombifrons* only can be found in the upper reaches of the Yarkand River in the Kashgar area, Yurungkax River, and Keriya River in the Hotan area at present, and no distribution report for Balkhash Lake has been published in other literature sources. We have not as yet been able to source a sample of *T. bombifrons* from Balkhash Lake, and cannot access any voucher information from previous reports [17]. In this study, we acquired a sample of *T. bombifrons* from the Yurungkax River and confirmed that its morphological characteristics are consistent with the description given in the literature (Figure 1). We wanted to compare the variation in the mitochondrial genome sequence, structure, and gene content in these two mitogenomes of *T. bombifrons* (NC_027189 and this study). In their study, a phylogenetic tree was reconstructed with only eight *Triplophysa* species, which placed *T. bombifrons* as a closed species with *T. strauchii*. With the rapid development of sequence technology, sequence costs have dropped dramatically, and sequencing read length and accuracy continue to improve [22,23]. High-fidelity (HiFi) reads in single-molecule sequences overcome the disadvantages of short-read sequencing technologies and can thereby bring us more accurate mitogenome information [24].

In this study, we report the complete mitogenome of *T. bombifrons*, assembled with HiFi reads of the Pacbio Sequel II system and DNBSEQ short-reads. We have carried out a comprehensive analysis of *Triplophysa* mitogenomes and reconstructed the phylogeny relationships of the genus *Triplophysa*, aiming to contribute the mitogenomic data of Nemacheilidae for future phylogenetic studies of the Cypriniformes.

## 2. Materials and Methods

### 2.1. Ethical Approval

The sample collection and animal experiments were conducted according to the regulations and guidelines for the care and use of laboratory animals and were approved by the Animal Care and Use Committee of Tarim University (protocol code TDDKYXF20220316).

### 2.2. Experimental Fish and Sampling

One adult *T. bombifrons* specimen was collected via nets in the Yurungkax River (37°6′39.6″ E, 79°54′46.8″ N), in the Hotan district of the Xinjiang Uygur Autonomous Regions, China. Voucher specimens were deposited at Tarim University (accession number GYQ2022030001, Xinyue Wang, 120050007@taru.edu.cn). The species and gender identification were determined by examining the dissected gonads. Pectoral fin clips were preserved in 75% ethanol and stored at −80 °C before DNA isolation.

### 2.3. DNA Isolation, Library Preparation, and Sequencing

The total genomic DNA was extracted using the TIANamp Genomic DNA Kit (TIANGEN, Beijing, China). The HiFi Library was prepared according to the manufacturer’s protocol. First, a 15 μg sample was selected and the SMRTbell^®^ Express Template Preparation Kit v2 was used to construct the SMRTbell library. The small DNA fragments were removed with BluePippin. The SMRTbell template was annealed with sequence primer, and the complex was bound by DNA polymerase. The library was sequenced on the Sequel II sequencing platform (Pacific Biosciences of California, Inc., Menlo Park, CA, USA). CCS (v.6.4.0) was used to generate the HiFi reads.

A total amount of 0.2 μg of DNA was used and the genomic DNA sample was fragmented into 350 bp fragments. The sequencing library was constructed following the manufacturer’s recommendations. The 5′ end of the library was phosphorylated and cyclized. The cyclized library was amplified by the rolling loop. Finally, the DNA nanospheres (DNB) were loaded into flowcell and then sequenced on the MGI DNBSEQ-T7 platform. In total, 20 Gb of short reads was generated. FastQC (v0.11.5) was used to qualify the sequence data-quality software [25]. Fastp (v 0.23) was used to filter low-quality reads, including those reads that contain more than 50% of bases with a Q-value of less than 2, and those reads that contain more than 5% of unknown nucleotides [26].

### 2.4. T. bombifrons Mitogenome Assembly and Annotation

The mitogenome of *T. bombifrons* was assembled with HiFi reads using the MitoHiFi (v2.2) pipeline [27]. The mitochondrial sequence of *Triplophysa angeli* (NC_065113.1) was used as the reference sequence since it is a closely related species to *T. bombifrons.* After completion of the nuclear genome assembly, the mitogenome sequence was extracted from the nuclear genome assembly using BLAST+ (v2.13.0) [28]. BWA (v.0.7.17) was used to align the short-reads from DNBSEQ-T7 to the new *T. bombifrons* mitogenome, then Pilon (v.1.24) was used for assembly polishing [29,30]. The mitogenome of *T. bombifrons* was annotated using Mitoz v3.4 [31]. The ORF Finder was used to determine 13 PCGs by comparing the reference mitogenome’s homologous sequences. In total, 22 tRNAs and 2 rRNAs were detected using MITOS [7]. Mitogenome maps were drawn using OGDRAW [32].

### 2.5. Sequence Analyses

Codon W was used to calculate the composition of the base, the pattern of codon usage, and the relative synonymous codon usage (RSCU). Patterns of nucleotide diversity (Pi), the non-synonymous (Ka) to synonymous rate (Ks) ratio of 13 PCGs among *Triplophysa* were conducted in DnaSP (v6.12.03). The sequence diversity of each PCG was estimated using sliding window analyses (window length ≤ 100 and step size = 25) in DnaSP. MEGA (v7.0) was used to estimate the genetic distances, using a Kimura-2 parameter (K2P) [33]. The number of single-nucleotide polymorphisms (SNPs) and indel sites was detected using the DnaSP software (v6.12.03) [34].

### 2.6. Phylogenetic Analyses

To clarify the phylogenetic relationships between *T. bombifrons* and other species in the *Triplophysa* genus, the 13 concatenated PCGs of *T. bombifrons* and other species available in GenBank (Table 1) were aligned using MAFFT, with default parameters [35]. The best-fit mode was calculated using the Akaike information criterion (AIC) in ModelFinder. Subsequently, the maximum-likelihood phylogenetic tree was reconstructed using IQ-TREE (v 2.1.2) with 1000 ultrafast bootstraps, under the GTR+F+R6 model [36,37].

## 3. Results and Discussion

### 3.1. Genome Structure and Base Composition

The newly complete mitogenome of *T. bombifrons* was identified as circular double-stranded molecules with a length of 16,568 bp, which exhibits striking similarity with other *Triplophysa* mitogenome sequences, differing from them between 24 bp and 113 bp, and 1 bp less than the previously published *T. bombifrons* mitogenome (Table 1). The mitogenome base composition is 27.46% A, 25.83% C, 18.58% G, and 28.13% T, with a slight AT bias (55.59%). Similar to other *Triplophysa* species, the mitogenomes of *T. bombifrons* contain 13 PCGs, 22 tRNAs, 2 rRNAs, and a putative control region (AT-rich region) (Figure 1, Table 2). The length of the 22 tRNAs ranged from 66 bp to 76 bp; tRNA^Cys^ was the shortest (67 bp), whereas tRNA^Lys^ was the longest (76 bp) in this study. The control region is 916 bp in length and is located between tRNA^Pro^ and tRNA^Phe^.

### 3.2. Description of Protein-Coding Genes (PCGs)

The majority strand (H-strand) encodes 28 genes, including *atp6*, *apt8*, *cox1*, *cox2*, *cox3*, *cob*, *nad1*, *nad2*, *nd3*, *nad4*, *nd4l*, *nad5*, *l-rRNA*, *s-rRNA*, *trnD*, *trnF*, *trnG*, *trnH*, *trnI*, *trnK*, *trnL*, *trnL*, *trnM*, *trnR*, *trnS*, *trnT*, *trnV*, and *trnW*. The remaining 9 genes (*nad6*, *trnQ*, *trnA*, *trnN*, *trnC*, *trnY*, *trnS*, *trnE*, and *trnP*) are encoded on the minority strand (L-strand). The gene order and gene orientation in this study are almost identical to other published studies of *Triplophysa* mitogenomes. However, compared with the previous report of the *T. bombifrons* mitogenome, the *trnE*, *trnS*, and *trnQ* genes are located on the L-strand in our study [17]. The typical start codons (ATG) were used in 12 PCGs, except for *cox1*, which starts with GTG. In total, 8 PCGs ended with the termination codon TAA (*cox1*, *cox2*, *atp8*, *apt6*, *cox3*, *nad4l*, *nad5*, and *cob*), and the remaining 5 PCGs terminated with TAG (Table 2). A similar arrangement and composition had been reported from other mitogenome studies in the genus *Nemacheilidae* [10,39].

 Both the expression levels of the genes and the stability of the mRNA were affected by the codon preference, providing evidence in analyzing the evolutionary patterns and phylogenetic relationship [40]. The 13 PCGs encoded a total of 5522 codons in the *T. bombifrons* mitogenome. Isoleucine, lysine, leucine, proline, phenylalanine, alanine, asparagine, and threonine acid were the codons with the highest usage, the usage rate accounting for 3.13%, 2.88%, 2.70%, 2.64%, 2.52%, 2.48%, and 2.44% in all codons, respectively. The arginines were the codons with the lowest usage and only accounted for 0.83% of all codons (Table 3). The stop codon (TAA) was the most frequently used in the PCGs of the *T. bombifrons* mitogenome in this study.

**Figure 2:**
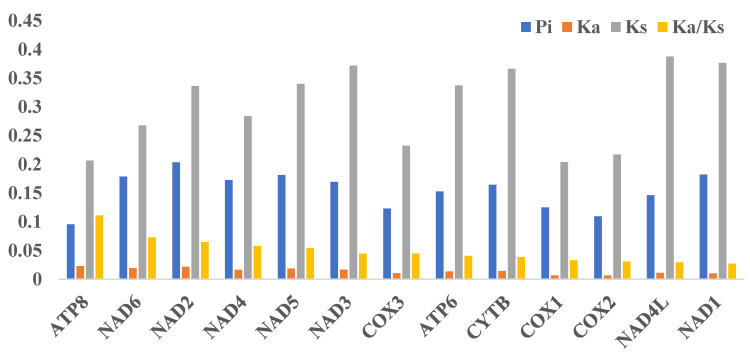
Genetic diversity and the Ka/Ks ratio of each PCG among the *Triplophysa* mitogenome.

As a significant indicator to identify molecular adaptation, the Ka/Ks ratio (ω) is widely used in phylogenetic analyses of molecular evolution [41]. The Ka/Ks (ω) values of the 13 PCGs were far lower than 1 (<0.12) (Figure 2), indicating that purifying selection was detected in these PCSs and these genes were suitable for reconstructing the phylogenetic relationship of the *Triplophysa* genus. The Ka/Ks of *atp8* (0.111), *nad6* (0.072), and *nad2* (0.065) are much higher than other PCGs, suggesting that these three PCGs had experienced more relaxed evolutionary pressure than other PGCs and retained more non-synonymous mutations in the genes. The lowest Ka/Ks ratio was found on the *nad1* gene, implying that the *nad1* gene had received the greatest evolutionary pressure. Mitochondrial DNA plays a vital role in encoding the essential components of the mitochondrial respiratory chain and its inheritance is strictly maternal, which makes deleterious mutations accumulate easily in the mitogenome. The nad genes are utilized as a co-substrate in non-redox reactions and play important roles in the signaling and regulatory pathways. The strong purifying selection detected in *nad1* helps to erase the deleterious mutations and makes the *nad1* gene a suitable molecular marker of phylogenetic analysis in the *Triplophysa* genus.

The aligned sequences of 13 PCGs of six *Triplophysa* mitogenomes were used to detect DNA polymorphism (Figure 2, Supplementary Table S2). The highest nucleotide diversity (Pi) was found in the *nad2* gene (0.203), followed by *nad1* (0.182), *nad5* (0.181), and *nad6* (0.178). The *cox3* (0.134), *cox1* (0.130), and *nad1* genes (0.129) have the lowest values. A similar pattern was also observed in terms of mean genetic distances (Table S3 in the Supplementary Materials). *Nad2*, *nad11*, *nad5*, and *nad6* genes showed high genetic distances with 0.24, 021, 0.21, and 0.21, whereas the *cox3*, *cox2*, and *atp8* genes exhibit lower genetic distances of 0.14, 0.12, and 0.10, respectively.

### 3.3. Sequence Divergence within T. bombifrons Mitogenomes

Comparing the two mitogenomes of *T. bombifrons* between our study and the previous report (NC_027189), 4.14% nucleotide dissimilarity (677 SNPs and 9 INDELs) had been found (Table S1 in the Supplementary Materials) [17]. In total, 550 SNPs were distributed widely among the 13 PCGs, the *nad5* gene (1839 bp) demonstrated a higher ratio (12.34%) than other PCGs relative to the size of the gene, whereas the *atp6* gene (684 bp) had the lowest ratio (0.58%). A similar result has been reported in *Branchinella kugenumaensis* mitogenomes, indicating that sequence divergence within the same species is a common phenomenon [20].

**Figure 3:**
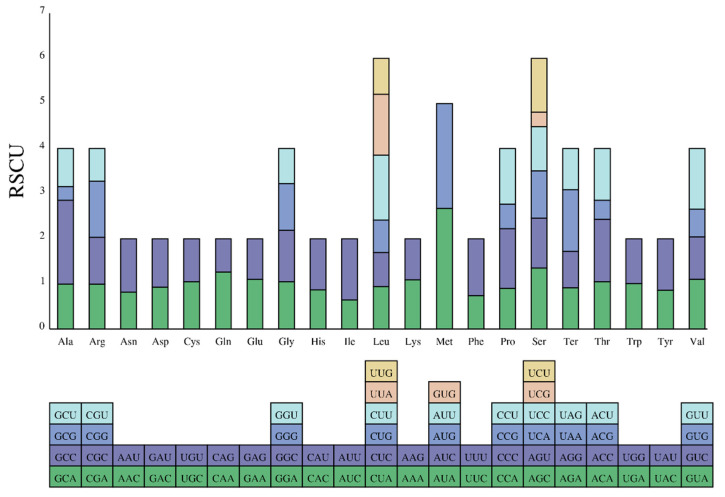
The codon content bar plot of the amino acids of 13 PCGs in the *T. bombifrons* mitogenome.

The similarity patterns of amino acid composition and synonymous codon usage were found in two *T. bombifrons* mitogenome sequences (Figure 3 and Supplementary Figure S1 in the Supplementary Materials). The analysis of RSCU showed that the 13 PGCs contain all codons. The five most frequently used codons in our study were Met (AUA), Met2 (AUG), Ala (GCC), Leu (CUU), and Thr (ACC), while in a previous report (NC_027189), they were Met (AUA), Met2 (AUG), Ala (GCC), Leu (CUU), and Ter (UAA) [17].

### 3.4. Phylogenetic Analyses

**Figure 4:**
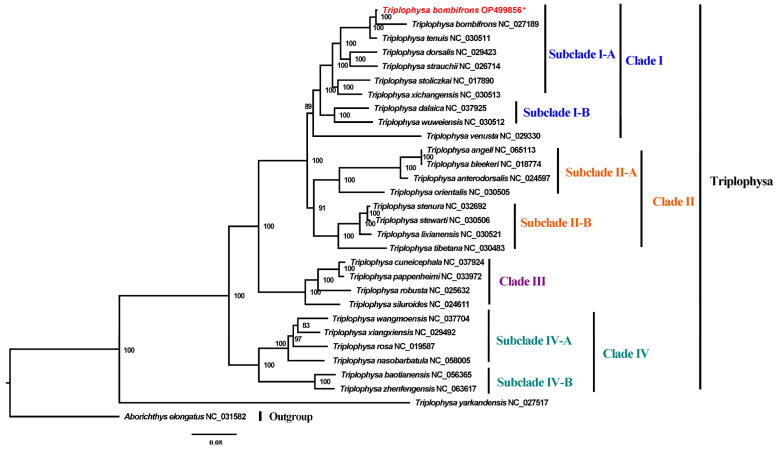
Phylogenetic relationship of 29 *Triplophysa* mitogenomes inferred by ML analyses, based on 13 PCGs. The number (%) on the branches indicates the maximum likelihood (ML) bootstrap support value. The *T. bombifrons* in this study was marked in red and asterick (*).

To ensure the reliability of the phylogenetic analyses, we downloaded all 28 mitogenomes of the *Triplophysa* species that have been characterized to date (28 October 2022) from the NCBI reference sequence (RefSeq) database [42]. The ML analyses showed *Triplophysa* contains 4 main clades (Clades I, II, III, and IV) (Figure 4). Clade I is divided into two subclades (I-A and I-B), with strong support in our phylogenetic reconstructions. The phylogenetic position of *T. bombifrons* indicated that it is the closest species to *T. tenuis* in subclade I-A, which was not reported in the previous *T. bombifrons* mitogenome report [17]. Subclade I-B encompassed *T. dalaica* and *T. wuweiensis*. Clade II comprised two subclades, both including 1 monophyly with 4 species. Species of *T. cuneicephala*, *T. pappenheimi*, *T. robusta*, and *T. siluroides* are included in Clade III. The remaining 6 species were divided into two subclades (Subclade IV-A and Subclade IV-B) and belong to Clade IV; this clade can be considered an ancestral group.

## 4. Conclusions

The results of the present study reported the complete mitogenome sequence of *T. bombifrons* using a hybrid assembly strategy, with PacBio HiFi read and DNBSEQ short-read sequence technologies. The structure of the evaluated *T. bombifrons* was identical to the mitogenome structure of the *Triplophysa* genus, including 13 PCGs, 22 tRNAs, 2 rRNAs, and one control region. Phylogenetic analyses based on the 13 PCGs strongly supported the idea that the genus *Triplophysa* should be divided into 4 main clades and demonstrated that *T. bombifrons* and *T. tenuis* are closely related species. The findings of this study will enrich resources of mitogenome in the genus *Triplophysa* and improve our knowledge of molecular characteristics in the Nemacheilidae family, providing a foundation for future study of population genetic and phylogenetic relationship in the Nemacheilidae family.

## Figures and Tables

**Table 1:** Detailed information of the mitogenome sequences from 29 *Triplophysa* and 1 *Aborichthys* species in this study.

Genus	Species	Size (bp)	Accession No	Resource
*Triplophysa*	*T. bombifrons*	16,568	OP499856	this study
	*T. bombifrons*	16,569	NC_027189	[17]
	*T. tenuis*	16,571	NC_030511	
	*T. dorsalis*	16,572	NC_029423	[15]
	*T. strauchii*	16,590	NC_026714	[17]
	*T. stoliczkai*	16,571	NC_017890	
	*T. xichangensis*	16,570	NC_030513	
	*T. dalaica*	16,569	NC_037925	
	*T. wuweiensis*	16,681	NC_030512	
	*T. venusta*	16,574	NC_029330	[16]
	*T. angeli*	16,569	NC_065113	
	*T. bleekeri*	16,568	NC_018774	[11]
	*T. anterodorsalis*	16,567	NC_024597	[10]
	*T. orientalis*	16,562	NC_030505	
	*T. stenura*	16,569	NC_032692	[12]
	*T. stewarti*	16,567	NC_030506	
	*T. lixianensis*	16,570	NC_030521	[7]
	*T. tibetana*	16,574	NC_030483	[9]
	*T. pappenheimi*	16,571	NC_037924	[6]
	*T. pappenheimi*	16,572	NC_033972	
	*T. robusta*	16,570	NC_025632	[19]
	*T. siluroides*	16,574	NC_024611	[5]
	*T. wangmoensis*	16,569	NC_037704	[8]
	*T. xiangxiensis*	16,598	NC_029492	[13]
	*T. rosa*	16,585	NC_019587	[14]
	*T. nasobarbatula*	16,605	NC_058005	[18]
	*T. baotianensis*	16,576	NC_056365	[4]
	*T. zhenfengensis*	16,564	NC_063617	[38]
	*T. yarkandensis*	16,574	NC_027517	[3]
*Aborichthys*	*A. elongatus*	16,544	NC_031582	

**Table 2:** Annotation of genes in the *T. bombifrons* mitogenome.

Locus	Start	Stop	Size (bp)	Start Coding	Stop Coding	Strand
*tRNA^Phe^*	1	69	69			H
*12S rRNA*	70	1017	948			H
*tRNA^Val^*	1020	1091	72			H
*16S rRNA*	1092	2768	1677			H
*tRNA^Leu^*	2769	2843	75			H
*nad1*	2844	3818	975	ATG	TAG	H
*tRNA^Ile^*	3826	3896	71			H
*tRNA^Gln^*	3895	3965	71			L
*tRNA^Met^*	3967	4035	69			H
*nad2*	4036	5082	1047	ATG	TAG	H
*tRNA^Trp^*	5081	5150	70			H
*tRNA^Ala^*	5153	5221	69			L
*tRNA^Asn^*	5223	5295	73			L
*tRNA^Cys^*	5327	5392	66			L
*tRNA^Tyr^*	5393	5460	68			L
*cox1*	5462	7012	1551	GTG	TAA	H
*tRNA^Ser^*	7013	7083	71			L
*tRNA^Asp^*	7086	7158	73			H
*cox2*	7172	7876	705	ATG	TAA	H
*tRNA^Lys^*	7863	7938	76			H
*atp8*	7940	8107	168	ATG	TAA	H
*atp6*	8098	8781	684	ATG	TAA	H
*cox3*	8781	9581	801	ATG	TAA	H
*tRNA^Gly^*	9565	9637	73			H
*nad3*	9638	9988	351	ATG	TAG	H
*tRNA^Arg^*	9987	10,056	70			H
*nad4l*	10,057	10,353	297	ATG	TAA	H
*nad4*	10,347	11,729	1383	ATG	TAG	H
*tRNA^His^*	11,729	11,798	70			H
*tRNA^Ser^*	11,799	11,866	68			H
*tRNA^Leu^*	11,868	11,940	73			H
*nad5*	11,941	13,779	1839	ATG	TAA	H
*nad6*	13,776	14,297	522	ATG	TAG	L
*tRNA^Glu^*	14,298	14,366	69			L
*cob*	14,372	15,532	1161	ATG	TAA	H
*tRNA^Thr^*	15,513	15,583	71			H
*tRNA^Pro^*	15,582	15,651	70			L

**Table 3:** Codon usage in the *T. bombifrons* mitogenome.

AminoAcid	Symbol	Codon	No.	Percent	RSCU
*	Ter	UAA	132	2.39%	1.3573
*	Ter	AGA	89	1.61%	0.9152
*	Ter	UAG	89	1.61%	0.9152
*	Ter	AGG	79	1.43%	0.8123
A	Ala	GCC	139	2.52%	1.8533
A	Ala	GCA	75	1.36%	1
A	Ala	GCU	63	1.14%	0.84
A	Ala	GCG	23	0.42%	0.3067
C	Cys	UGC	70	1.27%	1.0526
C	Cys	UGU	63	1.14%	0.9474
D	Asp	GAU	61	1.10%	1.0702
D	Asp	GAC	53	0.96%	0.9298
E	Glu	GAA	60	1.09%	1.1009
E	Glu	GAG	49	0.89%	0.8991
F	Phe	UUU	146	2.64%	1.2586
F	Phe	UUC	86	1.56%	0.7414
G	Gly	GGG	64	1.16%	1.0364
G	Gly	GGA	65	1.18%	1.0526
G	Gly	GGC	70	1.27%	1.1336
G	Gly	GGU	48	0.87%	0.7773
H	His	CAU	108	1.96%	1.1309
H	His	CAC	83	1.50%	0.8691
I	Ile	AUU	173	3.13%	1.3569
I	Ile	AUC	82	1.48%	0.6431
K	Lys	AAA	110	1.99%	1.0945
K	Lys	AAG	91	1.65%	0.9055
L	Leu	UUA	149	2.70%	1.3484
L	Leu	CUU	159	2.88%	1.4389
L	Leu	CUA	104	1.88%	0.9412
L	Leu	UUG	88	1.59%	0.7964
L	Leu	CUC	84	1.52%	0.7602
L	Leu	CUG	79	1.43%	0.7149
M	Met	AUG	85	1.54%	2.3224
M	Met	AUA	98	1.77%	2.6776
M	Met	AUC	0	0.00%	0
M	Met	AUU	0	0.00%	0
M	Met	GUG	0	0.00%	0
N	Asn	AAU	137	2.48%	1.1861
N	Asn	AAC	94	1.70%	0.8139
P	Pro	CCU	138	2.50%	1.2267
P	Pro	CCC	149	2.70%	1.3244
P	Pro	CCA	101	1.83%	0.8978
P	Pro	CCG	62	1.12%	0.5511
Q	Gln	CAA	108	1.96%	1.2632
Q	Gln	CAG	63	1.14%	0.7368
R	Arg	CGC	46	0.83%	1.0395
R	Arg	CGG	55	1.00%	1.2429
R	Arg	CGA	44	0.80%	0.9944
R	Arg	CGU	32	0.58%	0.7232
S	Ser	UCA	88	1.59%	1.0539
S	Ser	AGU	92	1.67%	1.1018
S	Ser	UCU	100	1.81%	1.1976
S	Ser	AGC	113	2.05%	1.3533
S	Ser	UCC	82	1.48%	0.982
S	Ser	UCG	26	0.47%	0.3114
T	Thr	ACA	103	1.87%	1.0537
T	Thr	ACU	112	2.03%	1.1458
T	Thr	ACC	135	2.44%	1.3811
T	Thr	ACG	41	0.74%	0.4194
V	Val	GUA	60	1.09%	1.106
V	Val	GUU	73	1.32%	1.3456
V	Val	GUC	51	0.92%	0.9401
V	Val	GUG	33	0.60%	0.6083
W	Trp	UGA	79	1.43%	1.0064
W	Trp	UGG	78	1.41%	0.9936
Y	Tyr	UAU	120	2.17%	1.1429
Y	Tyr	UAC	90	1.63%	0.8571

Note: “*” represent the stop codon.

## Data Availability

The genome sequence data that support the findings of this study are openly available in GenBank of NCBI at (https://www.ncbi.nlm.nih.gov/) under accession no OP499856 on 26 December 2022. The associated BioProject, SRA, and Bio-Sample numbers are PRJNA914502, SAMN32338863, SRR22839356 and SRR22839357, respectively.

## Supplementary Materials

The following supporting information can be downloaded at: https://zenodo.org/record/7299966 on 7 November 2022. Figure S1: Amino acid composition and relative synonymous codon usage in the mitogenomes of *T. bombifrons*; Table S1: Sequence divergence within *T. bombifrons* mitogenomes; Table S2: Nucleotide diversity, Ka, and Ks results of *Triplophysa*; Table S3: Genetic distances, based on 13 PCGs of *Triplophysa*.
